# Successful Management of a Broken Spinal Needle During Difficult Spinal Anesthesia: A Case Report

**DOI:** 10.7759/cureus.38822

**Published:** 2023-05-10

**Authors:** Thamer H Alsharif, Abdulkarim T Alanazi, Ahmed Deif, Hesham Aboueleneein

**Affiliations:** 1 School of Medicine, The Royal College of Surgeons in Ireland, Dublin, IRL; 2 Department of Surgery, Section of Neurosurgery, King Fahad Armed Forces Hospital, Jeddah, SAU

**Keywords:** epidural anesthesia, broken needle, spinal needle breakage, obstetrical anesthesia, spinal anesthesia

## Abstract

Spinal anesthesia is the most common mode of anesthesia utilized during caesarian sections worldwide. Despite its many advantages over general anesthesia in the pregnant cohort, uncommon and even catastrophic complications could occur due to patient-related, equipment-related, and procedure-related complications. One such uncommon event of a broken spinal needle during failed spinal anesthesia for a caesarian section and subsequent successful management is described here.

## Introduction

Spinal anesthesia was the first regional anesthetic technique used, and August Bier performed the first operation under spinal anesthesia in 1898 in Germany. Before this, the only existing methods of local anesthesia were infiltration anesthesia and topical anesthesia for the eyes [1].

In cesarean sections, spinal anesthesia is a widely used and recognized technique due to its benefits over general anesthesia. It is associated with low maternal discomfort, including a quick onset, a high success rate, and fewer adverse effects on the mother and fetus [2]. While the complications are low. Such as a backache, postural puncture headache, nausea, vomiting, hypotension, low-frequency hearing loss, and spinal hematoma [3-4].

Broken needles might lead to neurological complications depending on their location or if they are migrating toward the spinal canal. Potential complications include nerve damage (numbness, paraesthesia, or weakness), pain, infection, and CSF leakage [5]. The existing literature lacks reported cases of broken needles during central neuraxial blocks. However, over the last decade, more cases of broken needles in obstetric patients have been reported in the literature [6].

We report a case of a broken spinal needle during a failed, difficult spinal, which was removed early, avoiding serious complications in an obstetric patient.

## Case presentation

A 23-year-old primigravida, 38 weeks pregnant, presented to the ER with high blood pressure measured at 150/80 at home, associated with severe headache, no visual disturbance, and epigastric pain. Her initial blood reading was 148/72, and the follow-up reading was 146/90. Fundal height was +-40, and CTG was reactive.

On examination, she was conscious and alert, GCS 15/15, no lower limb swelling, and cervical OS closed. The urine dipstick was positive for +3 protein. She was admitted for pre-eclampsia, and informed consent was taken for an emergency Caesarean section. The patient was informed of the risks and benefits of receiving spinal anesthesia as well as what to expect throughout the procedure.

For the spinal anesthesia, the patient was in a sitting position, the skin was sterilized, and local anesthesia was administered. A metal introducer was used to insert a spinal needle, and resistance was met before the needle was withdrawn and advanced again. The anesthesiologist noticed the tip of the spinal needle broke on the fifth attempt after withdrawing the needle, which was confirmed by fluoroscopy. As the patient lacked adequate anesthesia, a decision was made for general anesthesia, and the surgery proceeded, and the fetus was delivered uneventfully.

**Figure 1:**
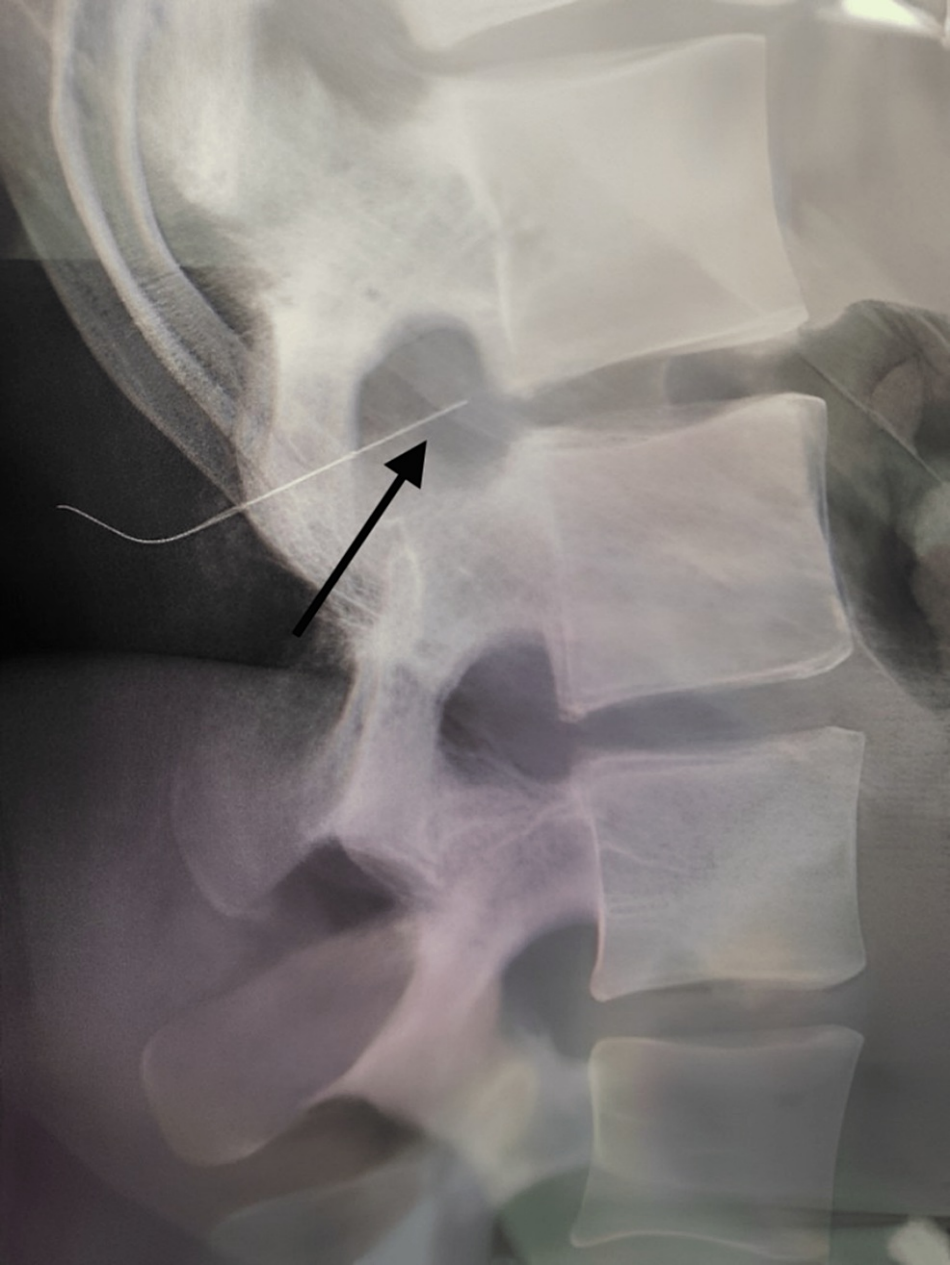
Lumbar spine X-ray lateral view showing needle tip opposite T12-L1

**Figure 2:**
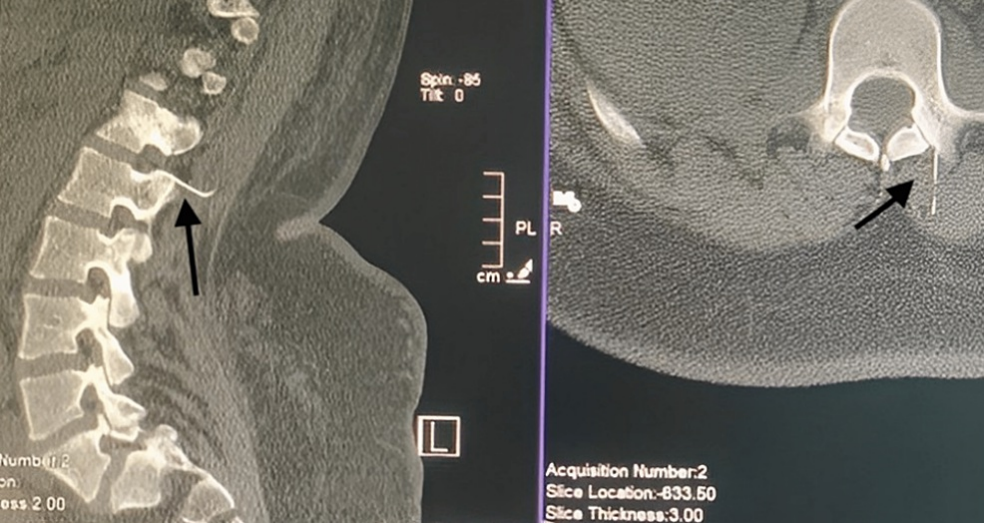
CT scan thoraco-lumbar spine axial and sagittal views show the broken needle tip at the left intervertebral foramen between T12-L1

The neurosurgery team recommended that the patient be discharged home until the C-section wound healed, after which surgery to remove the needle would be scheduled. Three weeks following the C-section. She came to the ER complaining of back pain with difficulty in flexion and extension of her back. On X-ray, the needle tip can be seen opposite T12-L1 (Figure 1). On a CT scan of the Lumbo-Sacral Spine, a broken spinal needle tip was observed on the left side, crossing the soft tissue of the back at the level of T12/L1, with the tip seen at the left intervertebral foramen, which was interpreted as a remnant of the needle that had been broken at the time of the spinal puncture for the cesarean section three weeks before (Figure 2).

To get the needle out, the exact location of the needle is identified intraoperatively using fluoroscopic guidance in the AP and lateral views. A paramedian incision was made, and muscle division was performed until the posterior end of the needle was visible and extracted. The closure was performed using 6.0 sutures and Dural after fluoroscopic confirmation of the removal of the needle and recording the absence of any fragments.

After the procedure, she was permitted to walk. The patient has surgical site pain, which has responded well to medications. She had no neurological impairment and was doing well. The wound was clear and dry, with no leaks when changing the dressing. She was discharged from the hospital, and the sutures were removed 12 days later. She resumed her normal activities without experiencing any side effects.

## Discussion

Spinal anesthesia is a safe technique that has been widely used and tested in the gynecological field, making it the first-line option in cesarean sections [7]. It can be performed more safely and effectively than general anesthesia, allowing for immediate bonding between a mother and her newborn [1].

However, if attempts at spinal anesthesia fail or a complication occurs, alternatives such as general anesthesia should be considered. Multiple failed attempts, emergency procedures, as in our case, and a high BMI are all risk factors for breaking a spinal needle [8]. In unsuccessful attempts, increased resistance during insertion, poor landmark differentiation, and concomitant needle deflection without introducer deflection was attributed. To reduce this risk, it is best not to continue or apply excessive force if there is abnormal resistance or bone contact during the administration, and it is also best not to withdraw the needle or change its direction through the introducer without mobilizing it first [9,10].

A broken needle can result in the needle fragment migrating and infecting the surrounding tissue. The risk of neurological complications is also dependent on the location of the needle, particularly if the needle is close to or moving toward the spinal canal. Nerve damage (numbness, paraesthesia, or weakness), pain, infection, and CSF leakage are all possible complications [5].

## Conclusions

Spinal anesthesia may be a straightforward procedure, but like all surgical procedures, it is not without danger and may occasionally present difficult consequences. One of them could be a broken needle while administering. The published studies state that a broken needle during administration is a relatively uncommon complication. In conclusion, surgical intervention to remove broken or lost needles is the preferred option, as it is effective and has a very reassuring outcome.
